# Pre-operative hand therapy management of Dupuytren’s disease: A systematic review

**DOI:** 10.1177/17589983241227162

**Published:** 2024-01-28

**Authors:** Joep Jannick Fernando, Christy Fowler, Tanya Graham, Kim Terry, Patricia Grocott, Fiona Sandford

**Affiliations:** 1 Department of Hand Therapy, 8945 Guy’s and St Thomas’ Hospitals NHS Trust, London, UK; 2 Florence Nightingale School of Nursing and Midwifery, 4616 King's College London, London, UK; 3 School of Biomedical Engineering & Imaging Sciences, King’s College London, London, UK

**Keywords:** Dupuytren’s, therapy, non operative, hand therapy

## Abstract

**Introduction:**

Dupuytren’s Disease is a fibroproliferative disorder of the hand, with a heterogenous pathogenesis, ranging from early-stage nodule development to late-stage digital contractures. Hand therapy intervention is not routinely provided pre-operatively. The objective of this systematic review was to explore the efficacy of hand therapy interventions provided for pre-operative Dupuytren’s Disease.

**Methods:**

A systematic review was undertaken of the databases CENTRAL, CINAHL, OVID Medline and OVID EMBASE, PubMed, BNI, Web of Science, with grey literature and reference searches conducted from database inception to April 2022, and confirmed in August 2023. Included studies required non-surgical intervention and outcome data on individuals with Dupuytren’s Disease who have not had surgical intervention. Two reviewers conducted the searches, independently assessed eligibility and completed methodological quality assessments. Data were summarised narratively.

**Results:**

Seventeen studies were selected for final inclusion. Interventions included Extracorporeal Shockwave Therapy (ESWT), Corticosteroid Injection (CSI), Splinting, Massage and Stretching, Ultrasound Therapy (US), Temperature Controlled High Energy Adjustable Laser (THEAL). ESWT positively maintained or improved pain, active range of motion (AROM), Disabilities of the Arm Shoulder, and Hand (DASH) scores, and grip strength. US positively maintained or improved ROM and grip. Splinting positively maintained or improved ROM, CSI positively improved nodule size. Cross Frictional Massage positively impacted AROM and THEAL improved pain and DASH scores.

**Conclusions:**

Outcomes from therapeutic interventions for pre-operative management of Dupuytren’s Disease were largely positive. However, there is a need for further high-quality research into these interventions to understand their full potential for the management of Dupuytren’s Disease.

## Introduction

Dupuytren’s Disease (DD) has a highly varied pathogenesis, with unpredictable progression. The location of diseased tissue impacts different structures (e.g. joint capsules, neurovascular bundles) altering clinical presentation. The prevalence of DD varies globally, between 0.2% and 56% of the population^1^ with the highest known rates in Western Europe.^2^ It has a gender predisposition towards men, reducing with age.^3^ In the UK more than five million people above 50 years of age are believed to be affected by the condition .^4^ Chiu and McFarlane ^5^ described three stages of DD (Table 1).

**Table 1.** Chui and McFarlane stages of Dupuytren’s Disease.

Stage	Name of stage	Characteristics of stage
1	Early disease	• Nodules in the palmar fascia with joint contracture
2	Active	• Increased nodular thickening • Associated joint contracture
3	Advanced	• Over 3 years of progressive joint contracture

Between 20% and 35% of people presenting with DD have progressive symptoms.^1,6,7^ For people with advanced disease, surgical intervention is deemed as the ‘gold standard’ management,^8^ and is the most widely provided treatment for this condition offered by the NHS.

Between 2007 and 2017, 121,488 people underwent Percutaneous Needle Fasciotomy (PNF), Limited Fasciectomy, or Dermofasciectomy in England.^9^ Almost one in four people were reported to have had complications after surgery^9^ including infection, injury to tendons or nerves, finger amputation, and systemic operative complications. Disease recurrence after surgical intervention ranges from 18% to 34%.^9^ A recent review^10^ estimates that approximately four million people in the UK with DD and experiencing functional limitation, do not meet the criteria to access currently provided intervention.

The dogma that non-surgical hand therapy intervention is ineffective^11^ is commonly cited as the reason to not provide treatment. With growing waitlists, significant operative risks, and the sizeable population experiencing disability with the disease who do not meet requirements for surgery, evaluation of non-surgical treatments is required. This systematic review aims to explore and evaluate non-surgical treatments, including advanced practice approaches that can be delivered by hand therapists for Dupuytren’s Disease.

## Methods

The review protocol was pre-registered with PROSPERO (CRD42022319007) and completed using PRISMA guidelines^12^ (see online supplementary material).

A systematic search was conducted of the following electronic databases: Cochrane Central Register of Controlled Trials (CENTRAL), British Nursing Index and Archive (BNI), OVID Medline, OVID EMBASE + EMBASE Classic, Web of Science, PubMed, CINAHL, from database inception to mid-April 2022. Search terms were selected after consultation with a health librarian provided in Table 2. Grey literature searches identified unpublished, non-peer-reviewed material, using ClinicalTrials.gov, WHO International Clinical Trial Registry Platform, Open Grey (discontinued in 2020), Easy DANS archive (post discontinuation of Open Grey), Google Scholar for first 50 hits utilising an incognito browser to avoid algorithmic bias. Grey literature search terms used were “Dupuytren’s” AND “Ultrasound” OR “splinting” OR “massage” OR “ESWT” OR “exercises” OR “steroids.”

**Table 2.** Search strategy.

Hand therapy treatments/Interventions	Dupuytren’s disease	Pre surgery	Body part
Hand physio* Hand physical therapist Hand occupational therapy Physio* Physical therapy Occupational therapy Rehab* EDUCATION Health literacy Self efficacy Patient understanding Splint* Orthosis Exercise Laser Shockwave Ultrasound Massage	Dupuy* Dupuytren* Viking disease Palmar fasci*	Conservative Non operative Early stages Nodules	Hand Finger* Thumb* Palm* Digit* PIP DIP MCP IP Proximal interphalangeal Distal interphalangeal Metacarpal phalangeal Interphalangeal

Footnotes; first row demonstrates key constructs and second row are search terms including MESH terms. BOOLEAN operators utilised the following structure; Search 1 – all terms OR (1) Search 2 – all terms OR (2) Search 3 – all terms OR (3) Search 4 – all terms OR (4) Search 5 – (1) AND (2) AND (3) AND (4) Search 6 – (2) AND (3) AND (4) Final – two lists merged search 5 and search 6

**Figure 1.**
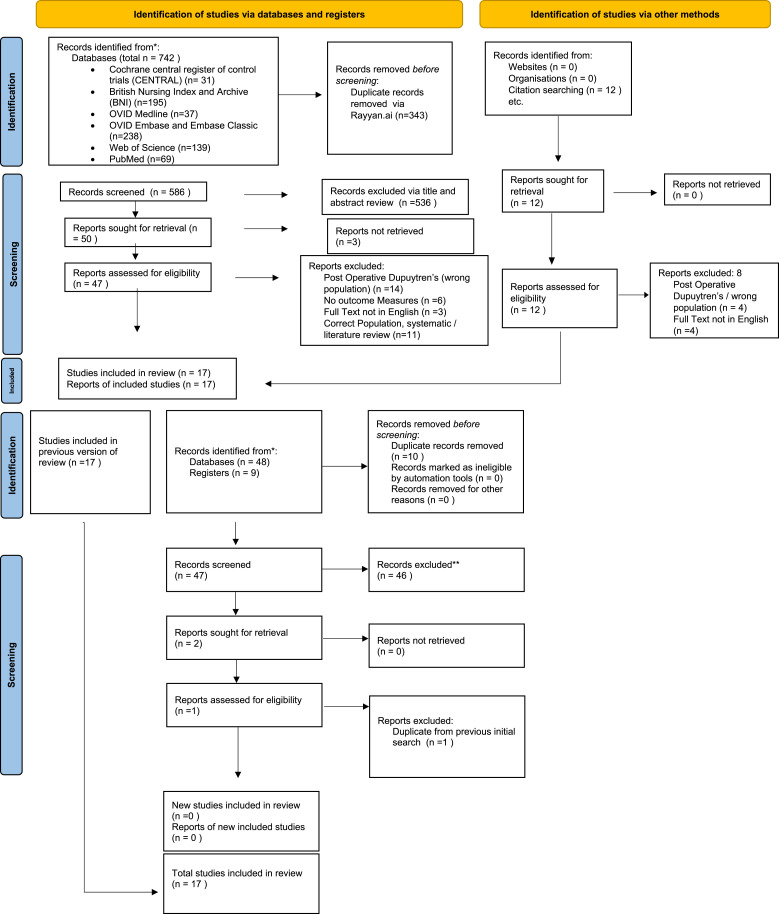
PRISMA flowchart 2022 and 2023.

All searches were conducted in the English language from database conception until October 2022 and repeated in August 2023 as per PRISMA Diagrams (Figure 1). Database searches were exported to reference management software, Rayyan.ai,^13^ whereby duplicates were removed without automation before screening. Title, abstract, and full-text screens were independently performed by JF and CF, against inclusion and exclusion criteria (Table 3). Adjudication was performed by TG (where consensus was not reached). All papers that had full-text screening had their reference list reviewed for relevant papers.

**Table 3.** Inclusion and Exclusion criteria.

Inclusion criteria	Exclusion criteria
• Studies with conservative (non invasive) treatments on patients’ with Dupuytren’s disease who have not undergone operative management • Any study design and outcomes • Reports available in English language or with freely available English translation	• Studies detailing operative management on patients’ with Dupuytren’s disease • Conservative treatments for post operative management of Dupuytren’s disease • Patients’ with other known neurological conditions affecting the hand • Patients under the age of 18 • Studies without outcome measures • Systematic reviews or literature reviews • Treatment of CCH or radiotherapy

Data were extracted into a table including study (author and year), type of study, sample size, population demographics (age, sex, grading/stage of disease), interventions, and outcomes, timescales and study limitations.

Each study was assessed for quality and risk of bias using the Joanna Briggs Institute (JBI) checklist for Case Report,^14^ JBI checklist for Case Series,^15^ and JBI checklist for randomised controlled trials^16^ (Table 4).

**Table 4.** JBI scores for all included papers.

Authors	Study type	Therapeutic modality	JBI score
Christie et al.,2011	Case report	CFM & stretch	7/8
Brunelli et al., 2020	Case report	ESWT	8/8
Mah and Branson, 2019	Case report	CSI	6/8
Taheri et al., 2021	Case report	ESWT	6/8
Ketchum and Donahue, 2000	Case series	CSI	3/10
Larocerie-Salgado and Davidson, 2010	Case series	Splinting	7/10
Aykut et al., 2018	Case series	ESWT	2/10
AbdulSalam et al., 2018	Case series	ESWT	8/10
Ball and Nanchahal, 2002	Case series	Splinting	5/10
Yin et al., 2016	Case series	Steroid	6/10
Markham and Wood, 1980	Case series	Ultrasound	7/10
Stiles, 1966	Case series	Ultrasound	0/10
Taheri et al., 2022	Case series	CSI	7/10
Brauns et al., 2016	RCT	Splinting	12/13
Knobloch et al., 2021	RCT	ESWT	9/13
Saad et al., 2021	RCT	ESWT	5/13
Notarnicola et al., 2017	RCT	ESWT/Ultrasound/stretch &massage	12/13

## Results

Seventeen studies were included: four case reports, nine case series and four randomised controlled trials. All studies were peer-reviewed, with no studies included from grey literature sources as per PRISMA diagrams (Figure 1).

After a review of study quality and risk of bias, there was an initial agreement rate of 69% (114/166). All disagreements were resolved through discussion or adjudication with a third reviewer if an agreement was not met. Disagreements were mostly related to a lack of information provided within the study papers.

Quality scores ranged across report type: case reports scored 6-8/8, case series scored 0–8/10, and randomised controlled trials scored 5–12/13. See Table 4 for a summary of all scores and the online supplementary material for in-depth scoring.

### Study characteristics

Studies were published between 1966 and 2022, in North America (three), Asia (five), Africa (one), Australia (one), and Europe (seven). Six interventions were identified: Extracorporeal Shockwave Therapy,^17–24^ Corticosteroid Injection,^25–27^ Splinting,^28–30^ Massage and Stretching,^21,31^ Ultrasound Therapy,^32,33^ Temperature Controlled High Energy Adjustable Laser.^21^

All studies recruited participants with stage 1 and stage 2 DD without significant digital contractures (which would qualify for a higher Chiu and McFarlane^5^ rating as per Table 1). There was significant heterogeneity amongst studies, therefore a narrative synthesis of interventions was performed. See online supplementary file I for study demographics.

### Electromagnetic high-energetic extracorporeal shockwave (ESWT)

Eight included studies evaluated the effect of ESWT on Dupuytren’s Disease.^17–24^ The studies were two case reports (quality 6–8/8), three case series (quality 2-8/10), and three (randomised) controlled trials (quality 5–12/13).

ESWT study protocols used varied parameters (Hz, pulse, session duration and frequency). Seven studies exclusively utilised ESWT. Saad et al.,^22^ compared whether the addition of ESWT to a traditional physical therapy protocol (ultrasound, massage, stretching, and splinting) improved participant outcomes.

Five studies evaluated pain (associated with early-stage DD) with the Visual Analogue Scale (VAS). No studies stated whether pain was assessed at rest or during function. Pain decreased from initial to final assessments in four of the five studies. In the remaining study^18^ pain returned to baseline levels. In the RCT by Knobloch et al,^20^ the control group receiving placebo ESWT had increased pain post-intervention.

Two RCT’s measured participant satisfaction with symptoms post-intervention. Knobloch et al^20^ found that participant satisfaction with symptom improvement was greater in the intervention groups. Notarnicola^21^ measured satisfaction (Roles and Maudsley score) with improvements in pain and decreases in limitation of activity at 3 months post-intervention.

All participants (including those not receiving ESWT) in Saad et al^22^ had a statistically significant increase in grip strength, with a greater increase in those receiving ESWT. No significant change in grip strength was identified in other studies.^17,20,22^ Seven studies recorded participant-reported outcome measures (PROM). Positive improvements in PROMS were observed in most studies. Whilst this improvement was shown at first follow up, this improvement in scores did not continue in longer-term follow-ups. Only Brunelli et al.,^19^ showed clinically significant improvement in PROM change scores.

Range of motion (ROM) improved post-ESWT in the four studies in which it was measured.^19,21,23,24^ Taheri’s et al.,^23^ single participant case report demonstrated an improvement in range of motion to ‘normal extension’ post-intervention. The case series by Taheri et al.,^24^ demonstrated statistically significant improvement in the extension range up to 14 weeks post-intervention. Notarnicola et al.,^21^ showed a statistically significant increase in total extension at the end of treatment and at 1-month post completion of treatment. Aykut et al^18^ reported the tabletop test turned negative in 16/23 participants.

### Cortico-steroid injection

Three studies investigated the outcomes of CSI for early-stage (Table 1) Dupuytren’s Disease.^25–27^ One was a case report (quality 6/8), with the remainder being case series (quality 3–6/10).

Ketchum & Donahue^25^ reported on a 4-years retrospective window of their 30-years practice of intralesional injections, however several participants in the series had received injections before the study period. Follow-up data was included for all injections, allowing the authors to have a follow-up period ranging from “30 months to 27 years”.^25^ ^p1158^

All studies reported a reduction in nodule size as the main outcome following injection. Yin et al^26^ used ultrasound by a single radiographer measuring maximal diameters of the nodules before and following injection, and clinical photography for qualitative review. Quantitative data demonstrated a statistically significant reduction in nodule diameter between injection at 6-months review and at the final review, with an “average size reduction of 56% at final review”.^26^ ^p680^

Yin et al.,^26^ reported 6% of participants experienced further growth of a treated nodule within 3 years of initial injection, requiring a further course of three injections. Ketchum and Donahue^25^ reported 50% of participants experienced further growth of the treated nodules within 1 - 3 years,^25^ ^p1159^ and subsequently required further injection.

Yin et al.,^26^ did not report any adverse events from CSI. Ketchum & Donahue,^25^ reported transient depigmentation or temporary subcutaneous atrophy at the injection site in 50% of participants. By 6 months after the final injection “nearly all” had complete resolution of the atrophy or depigmentation. Ketchum and Donahue^25^ reported two spontaneous flexor tendon ruptures that occurred in their 30 years of practice, “but not in the window of this 4-years study”.^25^ ^p1160^ The authors stated that the recommended practice of 6 months of respite between courses of injection was not followed with these two participants.

### Therapeutic ultrasound

Stiles^32^, and Markham & Wood^33^, studied the use of ultrasonic therapy, now commonly known as ultrasound therapy, with varying parameters (watts, pulse, treatment heads) and number of treatment sessions. Studies are historical, being published more than 40 years ago, with no current studies on the use of this modality. Both studies being case series had a quality ranging from 0 to 7/10.

During treatment in both studies, the clinicians extended the number of treatment sessions when authors identified positive improvements in outcomes. Across both studies, there was no correlation between the number of treatments and outcomes.

Both studies utilised a therapeutic adjunct, Markham and Wood^33^ referring to physiotherapy (Maitland Mobilisation, passive stretching, extensor tendon strengthening), while Stiles^32^ recommended participant-led passive stretching and for some participants, night splinting. It is difficult therefore to ascertain the true effect of ultrasound when removed from the other modalities.

The change in digital contracture was used as an outcome measure by Stiles^32^ and reported to have improved in one participant, unchanged in 11 participants, and worsened contracture in one participant. This improvement was not assessed with a formal range of motion measurement and as such is not reliable data.

Two participants from the study conducted by Markham and Wood^33^ were excluded by the reviewers on the premise that they were postoperative. Outcome data was unaggregated by study authors, therefore no statistical analysis was completed ex post facto. Non-operative participants demonstrated varying improvements including improved joint extension, mean hand span and grip strength at the 12-weeks follow-up. No statistical analysis has been conducted on the results by the study authors.

### Splinting

Three studies investigated the efficacy of splinting for Dupuytren’s Disease.^28–30^ Two studies were case series (quality 5–7/10) with one study being a randomised controlled trial (quality 12/13).

In all studies participants were reported as having stage 2 DD. Ball & Nanchahal^28^ and Larocerie-Salgado & Davidson^29^ studies included participants with mild to moderate MCPJ and or PIPJ contractures. In Braun’s et al^30^ RCT participants had greater extension deficits, when compared to the other studies. Braun^30^ included both “primary and recurrences”.^30^^p254^ Twelve of Brauns’s^30^ 30 participants had previously undergone surgical intervention, however all surgeries had taken “place at least 3 years before the initiation of the study protocol”^30^ ^p256^ and subsequently have been included in this systematic review as a result of the chronicity of the scarring process, and the likelihood that another further contracture at that stage would be related to Dupuytren’s tissue and not active scarring.

Bespoke static hand-based volar thermoplastic extension splints were used in two studies.^28,29^ Larocerie-Salgado & Davidson^29^ provided participants who did not tolerate bespoke tension or compression splints with either a circumferential finger-based splint or an LMB Spring Finger Extension Assist splint (North Coast Medical, Morgan Hill, CA). Brauns et al^30^ compared the efficacy of a “standardised tension orthosis”^30^ ^p254^ to “a newly designed compression orthosis with a silicone bed and Velcro strap”.^30^ ^p254^ The duration of advised splint wear varied from night-time^28,29^ to a total of 20 h per day.^30^

All three studies measured ROM. Brauns et al.,^30^ reported total active extension (TAE) of the affected digits but also measured isolated joints for subgroup analyses reporting a statistically significant reduction of TAE after 3 months in both the tension and compression groups, with no statistical differences between the two groups at 3 months. Brauns et al.,^30^ verified with a regression model that baseline characteristics such as history of surgery did not contribute to these results. Ball & Nanchahal^28^ did not perform any statistical analysis, however when the data were aggregated, there was a mean reduction of extension deficit of 9° at the metacarpophalangeal joint (MCPJ) and 24.3° at the proximal interphalangeal joint (PIPJ) at 4 months post-intervention. One participant reviewed at their final 6-month follow-up (before being lost to follow-up) presented with a maintenance of ROM. Two participants who reported to completely discontinue splint use at 6 months were recorded to have regressed by 10 and 13° at their PIPJ, while participants followed up at 24 months indicated maintenance improvement of extension by splinting for three to four nights per week .^28^ Larocerie-Salgado & Davidson ,^29^ demonstrated a statistically significant improvement or stabilisation in the degree of PIPJ extension between the time of initial consultation to follow-up. One participant in Larocerie-Salgado & Davidson’s study ^29^ who had been unable to tolerate the volar splint and subsequently provided with an LMB (North Coast Medical, Morgan Hill, CA) demonstrated worsening extension deficit by over 20° on three digits, proceeding to surgical intervention.

Brauns et al., ^30^ used a Visual Analogue Scale (VAS) to score aesthetic and functional satisfaction. In the compression Group 85%, and the tension Group 38%, self-rated themselves as very satisfied while wearing the splints from the start of treatment to 3-months timelines, however this was not statistically significant. Larocerie-Salgado & Davidson ^29^ noted a subjective softening and volume loss in the consistency of the fascial cords.

In studies that utilised splinting as a therapeutic adjunct, either there was no additional impact on the outcome or covariate analysis was not completed by authors in these two studies.^22,32^

### Cross-frictional massage and stretching

Two studies looked at the use of CFM and stretching to improve range of motion, pain, PROM scores, and morphological change to Dupuytren’s tissue. One was a case report with a quality score of 7/8 while the other was one arm of a randomised controlled trial with a quality score of 12/13.

Christie et al.,^31^ provided therapist-led CFM and stretching thrice weekly for 8 weeks, and Notarnicola et al.,^21^ taught participants self-directed stretches to be completed at home thrice daily for 2 weeks.

Range of motion was shown to improve with the addition of CFM compared to stretching alone. At 8 weeks post-intervention, the range of motion in little and ring fingers was reported to have improved by over 50% in the CFM and stretching group, in comparison to the control group of stretching alone which demonstrated no change or regression in comparison to pre-intervention baseline.^31^ In the stretching arm of Notarnicola’s^21^ RCT, the range of motion temporarily improved but returned to baseline 3 months post-intervention.

In Notarnicola’s RCT pain measured with VAS remained unchanged for the duration of the study period, while the DASH score reduced and remained reduced at a 3-months review. Ultrasound sonography did not demonstrate any morphological changes in either group post-intervention while subjective comparison using photography identified a “reduction in the size of nodules (and) visibility of contractile bands”, in the intervention group.

### THEAL – temperature controlled high energy adjustable laser therapy

Notarnicola et al ^21^ explored THEAL as one treatment arm of their randomised controlled trial with a quality score of 12/13. THEAL was delivered for 5 min, 5 times per week for 10 sessions, with consistent doses, the position of the arm during delivery, and direction of laser to the nodules. In comparison to the other treatment arms, THEAL had a statistically significant decrease in pain in the first month post-treatment and maintained lower scores at 3 months post-treatment. Patient outcomes were measured using the DASH and Roles and Maudsley score^21^ and showed statistically significant improvement for participants receiving THEAL. There was no change in extension deficit for participants in the THEAL arm of the RCT.

## Discussion

The widely held dogma is that surgical or pharmacological intervention is the optimal modality to treat DD.^11^ As highlighted by Nanchahal^10^ there are approximately four million people in the UK with symptoms of Dupuytren’s that never progress to meeting requirements for surgical intervention. Patients are often advised to allow the progressive nature of ‘typical’ DD to worsen, increasing symptoms, and decreasing range of motion in digits.^10^ The National Institute of Health and Care Excellence (NICE) recommends that patients who do not have contracture or significant loss of function do not require treatment. NICE recommends consideration of referral for a painful nodule before contracture development. Post-development of contracture or functional loss, the recommended treatments are surgery, steroid injection or radiotherapy.^34^ This review highlights potential treatment options for this patient cohort, that do report a level of impairment and disability but do not meet surgical criteria.

The studies in the review were able to demonstrate that splinting or stretching do not worsen disease progression outcomes as only one participant out of 49 participants who adhered to splinting protocols across three studies demonstrated regression in extension during intervention in the study period. Splinting is hypothesized to “act on the myofibroblasts”^30^ ^p254^ and this hypothesis of affecting tissues is applied across a range of conditions to justify splinting, so it is surprising that it is not equally encouraged in DD.

In the essential shift towards preventative healthcare models set out in the NHS Long Term Plan^35^ individualised patient care and encouraging maintenance of economic and social productivity in a growing ageing population, the need to provide alternatives to the surgical pathway is clear. It must be considered that in a known progressive disease, the positive outcomes of intervention may improve participants’ symptoms but also maintaining the current level of function and symptoms demonstrates the ability to delay the progression of the disease. This may avert the need for surgical intervention and associated complications,^9^ especially as participants were shown to have maintained improvement in symptoms.

While most studies demonstrate stabilisation of progression or improvement in symptoms, the delivery of the intervention and the period of follow-up and outcome monitoring was short and highly varied. These short follow-up periods of most studies are a limitation and will affect the ability of health providers being able to confidently deliver care that may only have a short-term effect, with unanswered questions of whether the interventions provided in the studies have an impact on the long-term progression of DD and a reduction in the requirement for surgical intervention. It is noted that all studies showed positive outcomes for patients during and immediately proceeding intervention. Longitudinal follow-up over years or even decades is essential.

Within each studied intervention, there remains a wide variation in treatment delivery protocol, including splinting approach (dorsal or volar, level of tension, duration of wear), ESWT (protocol, frequency, duration of intervention), CSI (type and concentration of steroid, injection approach), stretching (type of programme). This may be largely due to the lack of quality research to determine the most effective parameters of treatment or due to the varied clinical presentation and unpredictable progression of DD. Study outcomes taken were relevant to the symptoms targeted by the treatment. Not one study included a comprehensive set of outcomes to measure all known symptoms or deficits. Study follow-ups were also highly varied, whereby a narrative synthesis of the outcomes was challenging. With the development and utility of a hand and wrist core outcome set (ICHOM Set of Patient-Centred Outcome Measures for Hand and Wrist Conditions)^36^ and the use of the recommended extended finger track, future comparative reviews should be completed with greater ease.

NICE does not recommend non-surgical treatment modalities discussed in this review, despite the previous systematic review by Ball and Nanchahal^37^ synthesising conservative interventions for DD in 2016 including pharmacological, radiotherapy and physical therapy. Currently, patients are left to proactively seek out interventions, outside of NICE guidelines, often not funded by the NHS, potentially widening the gap in healthcare inequalities across the UK. This review adds to the body of evidence including new interventions of THEAL, ESWT, and the inclusion of steroid injection, that could be delivered by appropriately trained hand therapists.

Many studies focused on the impact of a particular modality however, it should be noted that in many studies participants were receiving adjunctive hand therapy treatment. When considering the role of a hand therapist, the ability to offer a wide range of multimodal interventions rather than being solely ‘splint technicians’ or ‘exercise prescribers’ is essential. This systematic review highlights the evidence supporting the available range of modalities thereby enabling patient choice and encouraging shared decision-making models in practice. Further research would be beneficial to examine the combination of modalities and the effect that this may have on disease progression, especially given therapists’ ability to deliver multiple treatments within one session.

## Limitations

While there were no restrictions in the country of publication, our systematic review’s inclusion criteria only included reports available in the English language due to resource limitations.

Due to the diverse range of experience within the systematic review study team, being a mixture of clinicians and academics, a study protocol was defined and submitted to PROSPERO prior to undertaking the systematic review, defining our question and structure. While we included a large range of medical databases and grey literature databases, we did not search thesis repositories which may have included valuable small-scale studies. This was under the assumption that these results would have been included in the grey literature searches or the Google Scholar search.

Few studies used statistical analysis or objective blinded assessment, increasing the risk of bias and compromising the validity of results. Studies with a high risk of bias and weak evidence base (Table 4), as highlighted with the use of the JBI Quality and risk of bias screen, were not excluded. Between clinicians and academics, the initial consensus of agreement was 69%. While this was resolved through discussion, this may have been attributed to diverse backgrounds of experience between researchers and academics.

Reports were not excluded on the premise of our inclusion criteria, to widely evaluate non-surgical intervention outcomes on those with early to mid-stage DD. While we identified three RCTs, constituting level 1 evidence, most studies that met inclusion criteria were Level 4 and 5 (Case Series, Reports).

Most studies included only stage 1 and stage 2 disease, no subgroup or meta-analysis regarding the pathophysiology and histopathology of participants was performed. This reduced our capacity to distinguish the effects of interventions between different levels of severity, adding to the heterogeneity of the disease within what was calculated to be a defined group of participants.

The inclusion of participants who had previously had surgery in Braun’s^30^ RCT, could be seen as a limitation of this review. However, the use of a regression model by Brauns et al.,^30^ to analyse if prior surgery impacted that outcome showed that no difference in outcome was seen from these participants.

## Conclusion

This systematic review recognises the range of non-surgical treatment modalities for individuals with Dupuytren’s Disease in the early stages. The evidence for the majority of participants undergoing conservative modalities indicated maintenance or improvement in symptoms, however the strength of the evidence remains largely that of level 4 and 5, and further high-quality research is needed to see the long-term effect and cost of the interventions. The use of the core outcome set for any studies would allow for greater comparison of future studies.

## Supplemental Material

Supplemental Material - Pre-operative hand therapy management of Dupuytren’s disease: A systematic review Supplemental Material for Pre-operative hand therapy management of Dupuytren’s disease: A systematic review by Joep Jannick Fernando, Fowler Christy, Tanya Graham, Kim Terry, Patricia Grocott, and Fiona Sandford in Hand Therapy

Supplemental Material - Pre-operative hand therapy management of Dupuytren’s disease: A systematic review Supplemental Material for Pre-operative hand therapy management of Dupuytren’s disease: A systematic review by Joep Jannick Fernando, Fowler Christy, Tanya Graham, Kim Terry, Patricia Grocott, and Fiona Sandford in Hand Therapy
