# Supplementary material for: Pre-operative hand therapy management of Dupuytren’s disease: A systematic review
Source: Hand Ther. 2024 Jan 28;29(2):52–61. doi: 10.1177/17589983241227162 (PMC11143942; doi:10.1177/17589983241227162)
Supplement: Supplemental Material - Pre-operative hand therapy management of Dupuytren’s disease: A systematic review [file sj-pdf-1-hth-10.1177_17589983241227162.pdf]

Supplementary File I

Table 5a: Study characteristics for extracorporeal shockwave therapy

Author, Year (Country)	Total Cohort of DD patients (hands)	Sex (M:F)<br><br>Mean age	Dupuytren's Stage (Chui & McFarlane) / Symptoms	Study Type	Pain (VAS)	Satis-faction (VAS)	Grip Strength (JAMAR)	Patient Reported Outcome Measures	Range of Motion	Clinical Imaging	Disease Progression
Aykut et al, 2018 (Turkey)	23 (28)	17 male<br>6 female<br><br>51 years	Stage 1: <30 degree contracture at MCPJs	Case Report	*Timescales between 3 measurements not clear*<br>Mean Score 1st measurement: 2<br>2nd measurement: 0<br>final measurement: 2		*Timescales between 3 measurements not clear* Mean Score: 1st measurement: 37.3kg<br>2nd measurement: 38 kg<br>final measurement: 42kg	*Timescales between 3 measurements not clear.*<br><br>QUICK DASH median 1st measurement: 15<br>2nd measurement: 9.1<br>final measurement 11.4<br><br>MAYO wrist score median: 1st measurement: 65<br>2nd measurement: 75<br>final measurement: 77.5	Table top test result turned negative for 16 of 23 patients		
Knobloch et al, 2021 (Germany)	52 (82)	32 male<br>20 female<br>52 years	Stage 1: painful nodules or cords without contracture	Randomised Controlled Trial	Treatment arm: PAIN VAS initial: 3.6 3 months post Rx: 1.9 6 months post Rx: 1.4 12 months: 1.7 and 18 months: 1.9<br>Control arm: Pain VAS initial: 2.2, 3 months post Rx: 3.4 6 months post treatment :3.4, 12 months post Rx: 3.4 18 months post Rx: 3.1	Improvement of symptoms 56% intervention group and 12% control.	No change in grip strength between two groups at initial or 18 months post intervention	DASH: Treatment arm: initial 12, 18 months post treatment 10 Control arm: initial 6, 18 months post Rx: 14 URAM : Treatment arm: Initial 3, 18 months post Rx 3, control arm initial 1, 18 months post Rx 3 . MHQ treatment arm initial 77 18 months post Rx 83, control group initial 79, 18 months post rx: 73			

Brunelli et al, 2020 Italy)	1 (1)	1 male<br>79 years	Stage 1: Nodules and total combined extension deficit 30 degrees of little and ring fingers	Case Series			DASH:<br>Initial: 32.1<br>1 week post Rx: 8.9<br>4 months post Rx 10.7<br>MHQ:<br>Initial 54<br>1 week post Rx: 76<br>4 months post Rx: 75	Total extension deficit LF & RF combined:<br>Initial: 30 degrees<br>1 week post Rx: 15 degrees<br>4 months post Rx: 15 degrees	
Abdulsalam et al, 2019 (Kuwait)	4 (7)	2 male 2 female<br>60 years	Stage 1: all patients had multiple nodules 50% also had flexion contracture (undefined angle)	Case Series	Pain Mean Score:<br>Initial 8.7<br>8 weeks post Rx: 2.0 Tender Mean Score Initial: 8.5<br>8 weeks post Rx: 2.5	No significant change or deterioration notes	MHQ: No significant change or deterioration noted		Ultrasonography noted similar echogenicity of nodules in all patients and decrease in size of nodules of only one patient
Taheri et al., 2022 (Iran)	20 (32)	11 male<br>9 female<br>66.6 years	Stage 1 or 2: flexion contracture of ≤15° in the PIP joint, flexion contracture of ≤30° in the MCP joint	Case Series	Mean score<br>Initial: 5.4.<br>6 weeks post Rx: 5.05 14 weeks post Rx: 4.85		DASH mean score:<br>Initial: 60.35<br>6 weeks post Rx: 52.95<br>14 weeks post Rx: 47.1	MCPJ extension deficit (mean)Initial:24.1<br>6 weeks post Rx: 21.75<br>14 weeks post Rx: 19.7	

Taheri et al., 2021 (Iran)	1 (2)	1 male<br>64 years	Stage 2: nodule and MCPJ extension deficit of 30 degrees	Case Report				QuickDASH Initial 24 to 1 week after completion of Rx: 18	Initial: 30 degree MCPJ restriction<br>1 week post completion of Rx: "normal"	
Notarnicola., 2017 (Italy)	45 patients (15 in each treatment arm)	32 male<br>13 female in entire study (unable to comment on M:F ratio in each arm)<br>63.4 years	Stage 1: Nodules and palpable cords affecting MCPJ or PIPJ	Prospective randomised study	Pre Rx: 4.0<br>15 days post Rx: 3.0 1 month post Rx 3.0 3 months post Rx: 3.0	Roles and Maudsley Satisfaction (median):<br>15 days post Rx: 3.0 1 month post Rx: 3.0 3 months post Rx: 2.0		DASH median score:<br>Pre Rx: 40<br>15 days post Rx: 40<br>1 month post Rx: 33<br>3 months post Rx: 30	Mean extension deficit (degrees)<br>Pre Rx: 50<br>15 days post Rx: 40<br>1 month post Rx: 20<br>3 months post Rx: 20	No results provided beyond 3 months post Rx
Saad et al., 2021 (Egypt)	30 (30)	Group A<br>14 Male<br>1 female<br>Group B<br>13 Male<br>2 Female	Stage 1: <30 degree contracture at MCPJs	(Randomised) Controlled Trial			Mean Grip Strength Intervention Group: Pre Rx: 15.73lb 6 weeks post Rx: 20.46 lb.<br>Control Group: Pre Rx: 16.13lb<br>6 weeks post Rx: 17.86lb	Modified Arabic Quick-DASH (mean score).<br>Intervention Group<br>Pre Rx: 26.73<br>6 weeks post Rx: 17.46<br>Control Group<br>Pre Rx: 25.06<br>6 weeks post Rx: 22.26		No results provided beyond 6 weeks post Rx

Table 5b: Study characteristics for Corticosteroid Injection (CSI)

Author, Year (Country)	Total Cohort of DD patients (hands)	Sex (M:F)<br><br>Mean age	Dupuytren's Stage (Chui & McFarlane) / Symptoms	Study Type	Pain (VAS)	Satis-faction (VAS)	Grip Strength (JAMAR)	Patient Reported Outcome Measures	Range of Motion	Clinical Imaging	Disease Progression
Ketchum and Donahue, 2000 (USA)	63 (75)	38Male 25Female 55 years	stage 1 or 2: all had nodules, nil had more than 15 extension deficit at MCPJ, nil had PIPJ extension deficit	Case Series						Photography of hands: 97% of the hands demonstrated 60% to 80% regression of the nodules with softening and flattening of the nodules.	50% of patients did experience are activation of disease in the nodules 1 to 3 years after the last injection, necessitating one or more injections. One patient opted for surgery as degree of FFD reached 40 degrees
Yin et al, 2016 (Taiwan)	37 (49)	28 Male 9 Female 72 years	Stage 1: Minimal cord, nodule, no flexion contracture	Case Series						Average nodule size (measured via ultrasonography) before Rx: 9mm<br>6 months post Rx: 5mm<br>5 years post Rx: 3mm	

Mah and Branson, 2019 (Australia)	1 (1)	1 Female 26 years	Stage 2: nodule, cord contracture at MCPJ	Case Report	Patient reported reduction in irritability post injection	Patient reports reduced size of chord and nodule					Patient reported no persisting cord contracture post injection

Table 5c: Study characteristics for Ultrasound

Author, Year (Country)	Total Cohort of DD patients (hands) [fingers]	Sex (M:F)<br><br>Mean age	Dupuytrens Stage (Chui & McFarlane) / Symptoms	Study Type	Pain (VAS)	Nodule Size	Grip Strength (JAMAR)	Span	Range of Motion	Disease Progression
Markham and Wood, 1980 (UK)	6 (7)	6 male 0 female	Stage 2: <30 degrees joint contracture, nodules	Case Series			(Initial, 4-12 Weeks) Mean grip improvement of 2.3kg	(Initial, 4-12 Weeks) Mean span improvement on 2cm	(Initial, 4-12 Weeks) Improvement in joint extension at: MCPJ 5 - 40 degrees PIPJ 2 - 15 degrees DIPJ 0 - 3 degrees	
PJ Styles, 1966 (London, UK)	8 (13)[22]			Case Series		(Initial, 4-12 Weeks) No improvement with nodular thickening. One patient thickening increased with a rapidly progressing contracture.			(Initial, 4-12 Weeks) Digital contracture improved 'slightly' in one patient. No improvement with nodular thickening. One patient thickening increased with a rapidly progressing contracture.	

Table 5d: Study characteristics for Massage and Stretching

Author, Year (Country)	Total Cohort of DD patients (hands)	Sex (M:F) Mean age	Dupuytrens Stage (Chui & McFarlane) / Symptoms	Study Type	Pain (VAS)	Nodule Size	PROMS (Patient Reported Outcome Measures)	Range of Motion	Disease Progression
Christie et al., 2011 (Canada)	1 (1)	1 male	<15 degrees joint contracture, nodules	Case Report		(Initial, 8 weeks, 16 weeks) Photography - subjective improvement in nodule size, wrinkling and visibility of contractile bands. Ultrasound sonography - nil changes from pre to post intervention		(Initial, 8 weeks) CFM with stretching: Active extension improvement of 11.5% (ring fingers) and 57.1% (little finger). Passive extension improvement of 77.8% and 30.0% respectively. Stretching: Active extension improvement of -1.9% (ring finger) and 0% (little finger). Passive extension improvement of 8.8% (ring finger) and 28.6% (little finger).	No regression of superficial improvements noted within 8 week period. At 16 week the 'majority' of ROM Improvement in CFM and Stretching > Stretching alone
Notarnicola et al, 2017 (Italy)	45 patients (15 in each treatment arm)	32 male 13 female in entire study	Stage 1: Nodules and palpable cords affecting MCPJ or PIPJ	Prospective randomised study	Mean score Pre Rx: 3.0 15 days post Rx: 3.0 1 month post Rx: 3.0 3 months post Rx: 3.0		DASH (mean score). Pre Rx: 48.3 15 days post Rx: 47.5 1 month post Rx: 47.5 3 months post Rx: 47.5	Mean extension deficit Pre Rx: 45.3 degrees 15 days post Rx: 42 degrees. 1 month post Rx: 41.7degrees..3 months post Rx: 42.7	

Table 5e: Study characteristics for THEAL (High Energy Laser Therapy)

Author, Year (Country)	Total Cohort of DD patients (hands)	Sex (M:F)<br><br>Mean age	Dupuytrens Stage (Chui & McFarlane) / Symptoms	Study Type	Pain (VAS)	Satisfaction (VAS)	Patient Reported Outcome Measures	Range of Motion	Disease Progression
Notarnicola et al, 2017 (Rome, Italy)	45 patients (15 in each treatment arm)	32 male 13 female in entire study (unable to comment on M:F ratio in each arm) 63.4 years	Stage 1: Nodules and palpable cords affecting MCPJ or PIPJ	Prospective randomised study	Mean score Pre Rx: 4.0 15 days post Rx: 2.0 1 month post Rx: 1.0 3 months post Rx: 1.0		DASH mean score Pre Rx: 40.8 15 days post Rx 40.0 1 month post Rx: 20.0 3 months post Rx: 20.0	Mean extension deficit Pre Rx:50 degrees. 15 days post Rx: 50 degrees 1 month post Rx: 50 degrees 3 months post Rx: 50 degrees	

Table 5f: Study characteristics for Splinting

Author, Year (Country)	Total Cohort of DD patients (hands)	Sex (M:F)	Dupuytrens Stage (Chui & McFarlane) / Symptoms	Study Type	Splint Type	Splint Wear Schedule	Length of Treatment	Satisfaction (VAS)	Functio n (VAS)	Change in ROM	Disease Progression
Ball and Nanchah al, 2002 (UK)	6 (7)	5 male and 1 female	Stage 2 Active / flexion contracture at MCP 2- 30 degrees or flexion contracture at PIPJ 27 - 86 degrees	Case Series	Hand based volar extensi on splint	Night wear	3 patients 24 months / 2 patients 6 months / 1 patient 4 months	Not measured	Not measur ed	Mean change of 9 degrees at MCPJ and 24.3 degrees at PIPJ of extension improvement at 4 months (most complete data set)	All patients maintained or improved their initial range of movement while wearing splints.
Larocerie -Salgado and Davidson, 2010 (Canada)	13 (13)	8 male and 5 female	Stage 2 Active / PIPJ flexion contracture between 15 and 60 degrees	Case Series	Hand based volar extensi on splint	Night wear (6-8 hours) PLUS pain free stretch in day and friction massage on nodule and cords	Average 12.6 months (SD: +-7.8; range: 2-27 months)	Not measured	Not measur ed	12/13 patients demonstrated improvement / stability of ROM: approximately 12.6 degrees (SD +/_5.1 degrees; range 5 - 25 degrees. 1 patient not able to tolerate splinting deteriorated	1 patient unable to tolerate splintng intervention deteriorated and pursued surgical intervention
Brauns et al, 2016 (Belgium)	30 (30)	21 male and 9 female	Stage 2 - 12 / 30 patients has previous surgery to the splinted finger > 3years prior to trial	Randomi sed Controlle d Trial	Levam e tension orthotic device	20 hours per day	3 months	4.8 to 7.0 points	6.3 to 8.0 points	Mean change in total active extension -32.4 degrees (95%CI:-39.6 to -25.1 degree) P<0.001	1 patient decided to switch treatment at 3/12 to CCH and discontinue splinting

Brauns et al, 2016 (Belgium)		Compression orthosis with silicone bed	20 hours per day	3 months	5.8 to 7.7 points	6.4 to 8.8	Mean change in total active extension -46.5degrees (95% CI: -62.2 to 30.8 degree) P<0.001
