# Supplementary material for: Pre-operative hand therapy management of Dupuytren’s disease: A systematic review
Source: Hand Ther. 2024 Jan 28;29(2):52–61. doi: 10.1177/17589983241227162 (PMC11143942; doi:10.1177/17589983241227162)
Supplement: Supplemental Material - Pre-operative hand therapy management of Dupuytren’s disease: A systematic review [file sj-pdf-2-hth-10.1177_17589983241227162.pdf]

Supplementary Material

RISK of Bias tables

JBI Case Report	*Christie et al.,2011*<br>*CFM & Stretch*<br>*7/8*	*Brunelli et al., 2020*<br>*ESWT*<br>*8/8*	*Mah and Branson, 2019*<br>*CSI*<br>*6/8*	*Taheri et al., 2021*<br>*ESWT*<br>*6/8*
Patient demographics	Yes	Yes	Yes	Yes
Patient history	Yes	Yes	Yes	Yes
Pre-intervention clinical condition	Yes	Yes	Yes	Yes
Diagnostic assessment methods	Yes	Yes	Yes	No
Intervention procedure	Yes	Yes	Unclear	Yes
Post-intervention clinical Condition	Yes	Yes	Yes	Yes
Adverse events identified and explained	No	Yes	No	No
Take away lessons highlighted	Yes	Yes	Yes	Yes

JBI Case Series	*Ketchum and Donahue, 2000*<br>*CSI*<br><br>*3/10*	*Lacorie-Salgado and Davidson, 2010*<br>*Splinting*<br><br>*7/10*	*Aykut et al, 2018*<br>*ESWT*<br><br>*2/10*	*AbdulSalam et al., 2018*<br>*ESWT*<br><br>*8/10*	*Ball and Nanchahal, 2002*<br>*Splinting*<br><br>*5/10*	*Yin et al., 2016*<br>*CSI*<br><br>*6/10*	*Markham and Wood, 1980*<br>*Ultrasound*<br><br>*7/10*	*Stiles, 1966*<br>*Ultrasound*<br><br>*0/10*	*Taheri et al., 2022*<br>*ESWT*<br><br>*7/10*
Clear inclusion criteria	Yes	Yes	Yes	Yes	Yes	Yes	Yes	No	Yes
Consistent measurement of condition	No	Unclear	Unclear	Yes	No	Yes	Unclear	No	Yes
Valid identification of condition	No	Unclear	Unclear	Yes	Unclear	Unclear	Yes	Unclear	Unclear
Consecutive participant inclusion	Unclear	Yes	Unclear	No	No	Unclear	Unclear	No	Unclear
Complete participant inclusion	Yes	Yes	Unclear	Unclear	Yes	Yes	Yes	Unclear	Yes
Clear reporting	No	Yes	No	Yes	Yes	Yes	Yes	No	Yes

demographics									
Clear reporting of participant clinical information	No	Yes	No	Yes	Yes	No	Yes	No	No
Clear reporting of outcomes	Yes	Yes	No	Yes	Yes	Yes	Yes	No	Yes
Clear reporting of sites/clinics demographics	No	No	No	Yes	No	No	Yes	No	Yes
Appropriate statistical Analysis	No	Yes	Yes	Yes	No	Yes	No	No	Yes

JBI RCT	*Brauns et al., 2016*<br>*Splinting*<br>*12/13*	*Knobloch et al., 2021*<br>*ESWT*<br>*9/13*	*Saad et al, 2021*<br>*ESWT*<br>*5/13*	*Notarnicola et al, 2017*<br>*ESWT, Stretching, THEAL*<br>*12/13*
True randomisation of participants	Yes	Yes	Unclear	Yes
Allocation groups	Yes	Yes	No	Yes

concealed				
Treatment groups similar at baseline	Yes	Yes	Yes	Yes
Participants blind to treatment assignment	Not Applicable	Unclear	No	Not Applicable
Clinicians blind to treatment group	Not Applicable	Unclear	No	Not Applicable
Assessors blind to treatment assignment	Yes	Unclear	No	Yes
Treatment groups identical other than intervention	Yes	Yes	No	Yes
Complete follow up	Yes	Yes	Yes	Yes
Analysis of participants in groups of randomisation	Yes	Yes	Yes	Yes
Consistent measurement of outcomes	Yes	Yes	Yes	Yes
Reliable measurement of outcomes	Unclear	Unclear	Unclear	Unclear

Appropriate statistical analysis	Yes	Yes	Yes	Yes
Appropriate trial design	Yes	Yes	No	Yes

PRISMA 2020 Checklist

Section and Topic	Item #	Checklist item	Location where item is reported
**TITLE**			
Title	1	Identify the report as a systematic review.	Title
**ABSTRACT**			
Abstract	2	See the PRISMA 2020 for Abstracts checklist.	Abstract
**INTRODUCTION**			
Rationale	3	Describe the rationale for the review in the context of existing knowledge.	Introduction
Objectives	4	Provide an explicit statement of the objective(s) or question(s) the review addresses.	Introduction
**METHODS**			
Eligibility criteria	5	Specify the inclusion and exclusion criteria for the review and how studies were grouped for the syntheses.	Table
Information sources	6	Specify all databases, registers, websites, organisations, reference lists and other sources searched or consulted to identify studies. Specify the date when each source was last searched or consulted.	Method and Table
Search strategy	7	Present the full search strategies for all databases, registers and websites, including any filters and limits used.	Table
Selection process	8	Specify the methods used to decide whether a study met the inclusion criteria of the review, including how many reviewers screened each record and each report retrieved, whether they worked independently, and if applicable, details of automation tools used in the process.	Method
Data collection process	9	Specify the methods used to collect data from reports, including how many reviewers collected data from each report, whether they worked independently, any processes for obtaining or confirming data from study investigators, and if applicable, details of automation tools used in the process.	Method
Data items	10a	List and define all outcomes for which data were sought. Specify whether all results that were compatible with each outcome domain in each study were sought (e.g. for all measures, time points, analyses), and if not, the methods used to decide which results to collect.	Table 5-9
	10b	List and define all other variables for which data were sought (e.g. participant and intervention characteristics, funding sources). Describe any assumptions made about any missing or unclear information.	Table 5-9
Study risk of bias assessment	11	Specify the methods used to assess risk of bias in the included studies, including details of the tool(s) used, how many reviewers assessed each study and whether they worked independently, and if applicable, details of automation tools used in the process.	Supplementary
Effect measures	12	Specify for each outcome the effect measure(s) (e.g. risk ratio, mean difference) used in the synthesis or presentation of results.	Narrative
Synthesis methods	13a	Describe the processes used to decide which studies were eligible for each synthesis (e.g. tabulating the study intervention characteristics and comparing against the planned groups for each synthesis (item #5)).	Method and Limitations
	13b	Describe any methods required to prepare the data for presentation or synthesis, such as handling of missing summary statistics, or data conversions.	Method
	13c	Describe any methods used to tabulate or visually display results of individual studies and syntheses.	Narrative
	13d	Describe any methods used to synthesize results and provide a rationale for the choice(s). If meta-analysis was performed, describe the model(s), method(s) to identify the presence and extent of statistical heterogeneity, and software package(s) used.	Narrative
	13e	Describe any methods used to explore possible causes of heterogeneity among study results (e.g. subgroup analysis, meta-regression).	Narrative
	13f	Describe any sensitivity analyses conducted to assess robustness of the synthesized results.	Narrative
Reporting bias assessment	14	Describe any methods used to assess risk of bias due to missing results in a synthesis (arising from reporting biases).	Table 4
Certainty assessment	15	Describe any methods used to assess certainty (or confidence) in the body of evidence for an outcome.	Table 4

PRISMA 2020 Checklist

Section and Topic	Item #	Checklist item	Location where item is reported
**RESULTS**			
Study selection	16a	Describe the results of the search and selection process, from the number of records identified in the search to the number of studies included in the review, ideally using a flow diagram.	Image 2 and 3
	16b	Cite studies that might appear to meet the inclusion criteria, but which were excluded, and explain why they were excluded.	Image 2 and 3
Study characteristics	17	Cite each included study and present its characteristics.	Table 5 - 9
Risk of bias in studies	18	Present assessments of risk of bias for each included study.	Table 5 - 9
Results of individual studies	19	For all outcomes, present, for each study: (a) summary statistics for each group (where appropriate) and (b) an effect estimate and its precision (e.g. confidence/credible interval), ideally using structured tables or plots.	Table 5 - 9
Results of syntheses	20a	For each synthesis, briefly summarise the characteristics and risk of bias among contributing studies.	Results
	20b	Present results of all statistical syntheses conducted. If meta-analysis was done, present for each the summary estimate and its precision (e.g. confidence/credible interval) and measures of statistical heterogeneity. If comparing groups, describe the direction of the effect.	Narrative
	20c	Present results of all investigations of possible causes of heterogeneity among study results.	Results and discussion
	20d	Present results of all sensitivity analyses conducted to assess the robustness of the synthesized results.	Narrative
Reporting biases	21	Present assessments of risk of bias due to missing results (arising from reporting biases) for each synthesis assessed.	Results and discussion
Certainty of evidence	22	Present assessments of certainty (or confidence) in the body of evidence for each outcome assessed.	Results, discussion, Limitations
**DISCUSSION**			
Discussion	23a	Provide a general interpretation of the results in the context of other evidence.	Discussion
	23b	Discuss any limitations of the evidence included in the review.	Limitations
	23c	Discuss any limitations of the review processes used.	Limitations
	23d	Discuss implications of the results for practice, policy, and future research.	Discussion and Conclusion
**OTHER INFORMATION**			
Registration and protocol	24a	Provide registration information for the review, including register name and registration number, or state that the review was not registered.	Introduction - PROSPERO
	24b	Indicate where the review protocol can be accessed, or state that a protocol was not prepared.	Introduction - PROSPERO
	24c	Describe and explain any amendments to information provided at registration or in the protocol.	Introduction - PROSPERO
Support	25	Describe sources of financial or non-financial support for the review, and the role of the funders or sponsors in the review.	Declarations
Competing interests	26	Declare any competing interests of review authors.	Declarations
Availability of data, code and other materials	27	Report which of the following are publicly available and where they can be found: template data collection forms; data extracted from included studies; data used for all analyses; analytic code; any other materials used in the review.	Material uploaded to SAGE

From: Page MJ, McKenzie JE, Bossuyt PM, Boutron I, Hoffmann TC, Mulrow CD, et al. The PRISMA 2020 statement: an updated guideline for reporting systematic reviews. BMJ 2021;372:n71. doi: 10.1136/bmj.n71

For more information, visit: http://www.prisma-statement.org/
